# The evolution of gene regulation

**DOI:** 10.7554/eLife.27291

**Published:** 2017-05-12

**Authors:** Veronica Hinman, Gregory Cary

**Affiliations:** 1 Department of Biological Sciences, Carnegie Mellon University, Pittsburgh, United Statesvhinman@andrew.cmu.edu; 1 Department of Biological Sciences, Carnegie Mellon University, Pittsburgh, United States

**Keywords:** *A. queenslandica*, evolution of multicellularity, histone modifications, cis-regulation, enhancers, gene expression, Other

## Abstract

The gene regulation mechanisms necessary for the development of complex multicellular animals have been found in sponges.

**Related research article** Gaiti F, Jindrich K, Fernandez-Valverde SL, Roper KE, Degnan BM, Tanurdžić M. 2017. Landscape of histone modifications in a sponge reveals the origin of animal *cis*-regulatory complexity. *eLife* **6**:e22194. doi: 10.7554/eLife.22194

The evolution of multicellular organisms from simple, single-cell organisms was a pivotal turning point in the history of life. Many different lineages of organisms, including animals, independently evolved multicellularity. One advantage of multicellularity is that cells can be programmed to perform particular biological roles. However, in order to assign cells to a specific role or fate, genes within certain tissues need to be activated at precise times during development. This process, which is known as spatiotemporal regulation, is orchestrated by various regulatory genes, including transcription factors and cell-signaling molecules.

A startling revelation in the field of evolutionary developmental biology was the discovery that the suite of regulatory genes that controls development in animals is ancient (Degnan et al., 2009; King et al., 2003). Since animals share many of the same regulatory genes, the immense diversity of the animal kingdom must be a result of how these genes are used during development. Knowing where and when specific genes are expressed is, therefore, critical for the study of animal development and evolution.

The DNA in a cell is usually wrapped around proteins called histones, which are packed tightly together to form a structure called chromatin (Figure 1A). The main purpose of histones is to condense DNA and to regulate chromatin (and, therefore, to influence gene regulation). Histones can bear certain chemical marks, which are a result of chemical changes to the structure of the histone, also known as post-translational modifications. The modifications can affect gene expression through various mechanisms, and can either activate or repress DNA regions (Zhou et al., 2011). These mechanisms presumably evolved along with the need for a system to control development in animals. Until recently, however, we have not known just how ancient these mechanisms of gene regulation are among animals.

**Figure 1.**
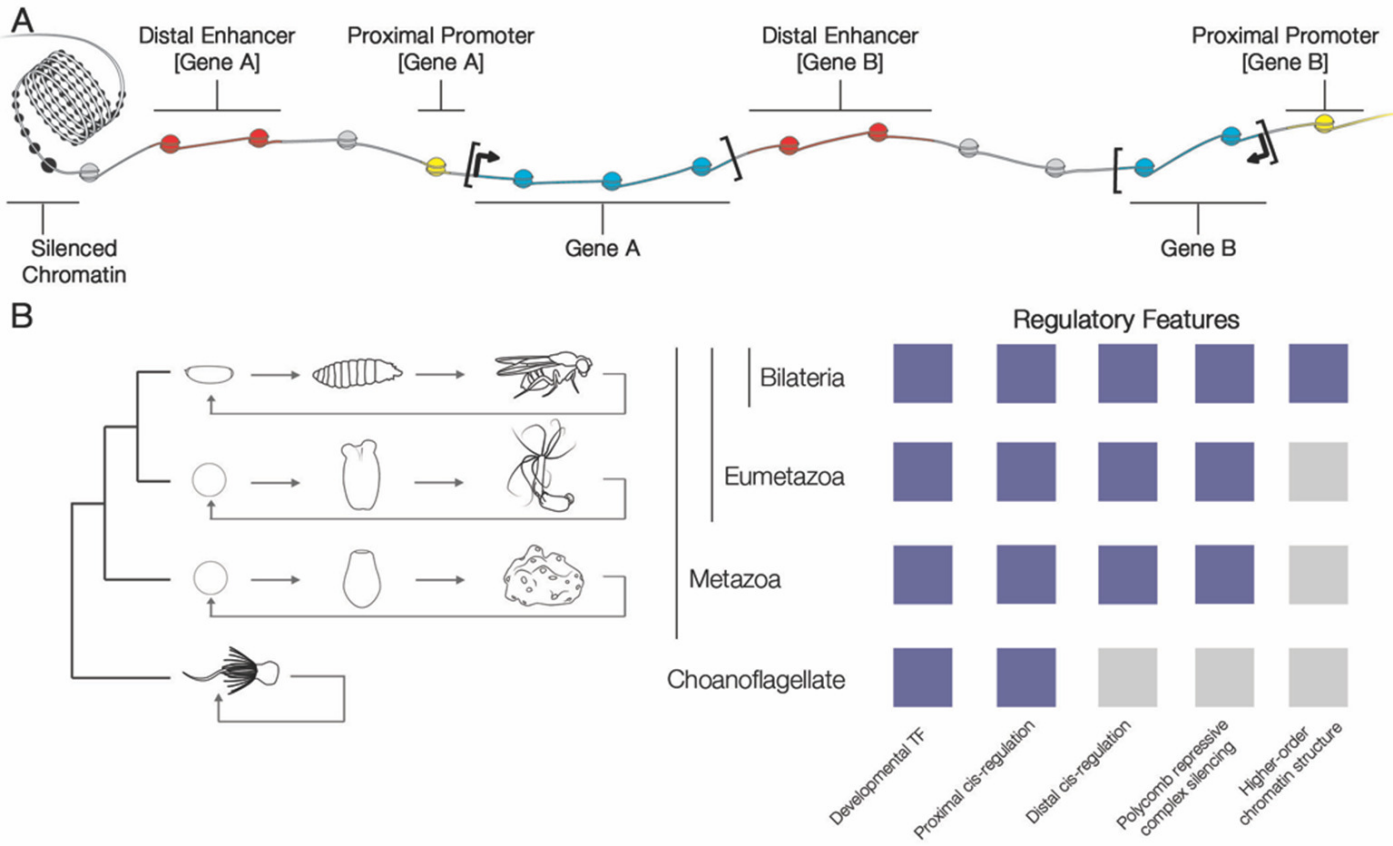
Evolution of genome regulation during animal evolution. (**A**) The DNA in cells is wrapped around proteins called histones (spheres) and which are then tightly packaged inside a structure called chromatin (left). The genes in the DNA cannot be expressed as proteins when the DNA is tightly packaged inside chromatin. In addition to genes (shown here in blue), the DNA contains *cis*-regulatory elements such as promoters (yellow) and enhancers (red), and the expression of a given gene is usually coordinated by a large number of these elements (although only two are shown for each gene here). In this example the fact that the distal element regulating gene B is adjacent to gene A is predicted to constrain the evolution of both genes. (**B**) The phylogenetic relationship of bilateria, eumetazoa, metazoa and choanoflagellates is shown (left), along with the genomic regulatory features found in each example species (right). All animals (metazoans) progress through development to multicellular adults, which distinguishes them from closely related unicellular organisms (such as the choanoflagellates). Bilateria, such as *Drosophila melanogaster*, have several regulatory features that are absent in related unicellular species. However, a number of these regulatory features have been characterized in basal eumetazoans (e.g. sea anemones) and now basal metazoans (e.g. *Amphimedon queenslandica*).

Now, in eLife, Milos Tanurdžić, Bernard Degnan and colleagues at the University of Queensland –﻿ including Federico Gaiti as first author –﻿ report that the regulatory landscape necessary for developing complex multicellular animals is present in the sponge *Amphimedon queenslandica *(Gaiti et al., 2017). Sponges diverged from other animals very early in the evolution of the metazoa (Figure 1B; Erwin et al., 2011), so common features between modern sponges and other animals are thought to have been present in the ancestor of all animals.

Gaiti et al. used a technique called ChIP-seq to determine the location of several post-translational modifications at different stages in the life of *Amphimedon*. In particular, they were interested in specific modifications associated with regulatory features that had previously only been identified in more complex animals. Gaiti et al. found evidence for the same kinds of regulation in *Amphimedon,* including chromatin repressors and distal *cis*-regulatory elements – regions of non-coding DNA that control the transcription of nearby genes. Overall, their results provide evidence that sponges use the same regulatory system found in more complex animals.

One of the modifications they studied repressed or silenced chromatin, and thus reduced or prevented the expression of certain genes. This particular modification involves a structure called the Polycomb Repressive Complex 2, which is a group of proteins that play an important role in cell development and can modify histones to silence chromatin. Gaiti et al. show that homologs of all parts of this complex, as well as transcription factors that target the complex to specific DNA sequences, exist in *Amphimedon*. Previous research has shown that the Polycomb Complex represses specific sets of genes that help cells to differentiate into specific cell types by silencing chromatin. As the silencing persists over several cell generations, a particular cell fate can be maintained over several cell cycles (Margueron and Reinberg, 2011). So, even after many cell divisions, a cell retains information that it needs to develop into a certain cell type. This kind of regulation is not found in related unicellular organisms, and the fact that it is found in *Amphimedon* suggests that 'cellular memory' is a feature common to all multicellular animals.

Gaiti et al. also examined the role of non-gene coding DNA elements in *Amphimedon*. Regulatory elements are classified based on their proximity to the gene they control, with proximal elements being adjacent to their genes and distal elements being farther away from the gene body (Levine et al., 2014). Distal *cis*-regulatory elements are critical for accurate spatiotemporal expression of the genes that regulate development in modern animals (Schwaiger et al., 2014). Gaiti et al. predicted the distal *cis-*regulatory elements in *Amphimedon* based on the combinations of histone modifications and position relative to transcribed genes. They discovered that these elements contained binding sites for transcription factors that are important for development and were similar to the *cis*-regulatory elements found in other animals.

Over time during evolution, most genes will move around relative to the position of other genes. However, it is thought that genes that remain adjacent to each other are somehow dependent on each other and share coordinated transcriptional regulation and gene expression. For example, the expression of one of the genes might depend on the *cis*-regulatory sequence near the neighbouring gene (e.g. Figure 1A). Indeed, previous research has shown that linked gene pairs that also share common *cis*-regulatory elements remain linked through evolution (Irimia et al., 2012). When Gaiti et al. looked at conserved, linked gene pairs in *Amphimedon*, they found that many did contain these predicted distal elements, supporting the results that these elements are indeed functional. These findings indicate that such regulatory elements are shared with all animals, but are absent in related single-cell species (Sebé-Pedrós et al., 2016).

It appears that a complex regulatory network – which includes the modification of histones that affect chromatin domains, distal *cis*-regulatory elements and a cellular memory mediated by the Polycomb Repressive Complex – is common to all multicellular animals, including sponges. However, it remains unclear when exactly, or how, these control programs evolved, and what other mechanisms contributed to the diversity and complexity of modern animals. Certain structural characteristics of chromatin that enable regulatory regions to communicate with more distant regions may be absent from *Amphemidon*, as some proteins that establish these interactions are only found in animals with bilateral symmetry (Heger et al., 2012). Nevertheless, the regulatory complexity required for the dynamic control of developmental gene expression had evolved by the time the sponges first diverged from the other animals, and therefore preceded the evolution of multicellular animal life.
